# In This Issue

**Published:** 1998

## ALCOHOL CONSUMPTION AMONG RACIAL/ETHNIC MINORITIES

Over the past decade, greater attention has been given to the study of alcohol use and its consequences among special populations in the United States, with particular emphasis on blacks, Hispanics, Asian-Americans, and Native Americans. Drs. Raul Caetano, Catherine L. Clark, and Tammy Tam report that such studies are particularly challenging because of the great diversity of racial/ethnic backgrounds and the variety of drinking patterns and practices found within each of those groups. For example, drinking rates differ considerably among Hispanics of Mexican, Puerto-Rican, and Cuban descent. Similar differences exist among Asian-Americans from various ethnic backgrounds and among members of the different Native American tribes. Accordingly, simple models that focus on only one factor to explain drinking behaviors cannot fully account for the wide range of consumption patterns observed in each group. (pp. 233–241)

## ALCOHOL CONSUMPTION IN INDIA, MEXICO, AND NIGERIA: A CROSS-CULTURAL COMPARISON

People’s attitudes toward alcohol vary substantially among different countries. For example, India, Mexico, and Nigeria are three countries that contain vastly different cultures and distinct drinking practices, report Drs. Linda A. Bennett, Carlos Campillo, C.R. Chandrashekar, and Oye Gureje. For example, overall alcohol consumption is low in India. Conversely, alcohol use is an integral part of adult social life in Nigeria. Mexican drinking practices fall between these two margins, with high rates of abstinence, particularly among women, but frequent and heavy drinking among those people who do consume alcohol. By comparing various cultures’ drinking practices and attitudes toward alcohol, researchers, clinicians, and policymakers will be able to develop more effective prevention and treatment strategies as well as alcohol-related policies that are tailored to fit the needs of the particular country. (pp. 243–252)

## AMERICAN INDIANS AND ALCOHOL

The high prevalence of alcohol use and its related problems among American Indians may stem from a variety of factors, ranging from the influence of the European colonizers to current social and cultural factors. Dr. Fred Beauvais summarizes research on alcohol use among American Indians and describes approaches to treating and preventing alcohol abuse and alcoholism in this population. According to Dr. Beauvais, inclusion of Native beliefs and approaches may prove useful in treating and preventing alcohol problems in American Indians. (pp. 253–259)

## DRINKING PATTERNS AND PROBLEMS AMONG AFRICAN-AMERICANS: RECENT FINDINGS

Investigations of alcohol use among African-Americans make up a small but growing area of alcohol research. Findings consistently reveal that although more African-Americans than whites abstain from drinking, similar levels of frequent heavy drinking are found in both groups. Dr. Rhonda Jones-Webb reviews recent research on the drinking patterns and drinking problems in African-American populations. The author also describes the limitations of existing research and offers directions for future studies. (pp. 260–264)

## ALCOHOL USE AMONG CUBAN-AMERICANS, MEXICAN-AMERICANS, AND PUERTO RICANS

Patterns of alcohol consumption among Hispanics in the United States vary considerably. In this article, Whitney M. Randolph and Drs. Christine Stroup-Benham, Sandra A. Black, and Kyriakos S. Markides compare drinking patterns among the three largest Hispanic subgroups in the United States—Cuban-Americans, Mexican-Americans, and Puerto Ricans. The authors show how this disparity in drinking patterns may be related to variations in the culture of origin, a result of the individual ethnic group’s merging into mainstream culture, or a combination of both factors. The authors note that future research should include previously studied groups as well as the growing numbers of Hispanics from Central America and other areas. (pp. 265–269)

## DRINKING PATTERNS AND DRINKING PROBLEMS AMONG ASIAN-AMERICANS AND PACIFIC ISLANDERS

People of Asian and Pacific Islander (API) descent are the fastest growing minority population in the United States. Dr. Kiyoko Makimoto reviews studies that have evaluated drinking behaviors and alcohol-related problems among APIs in recent years. Those studies found that APIs generally have lower rates of alcohol use and alcoholism than do other groups. Nevertheless, tremendous variability exists in the drinking behaviors of different API subgroups. For example, drinking and heavy-drinking rates are significantly higher among Japanese-Americans than among Chinese-Americans. Furthermore, many Southeast Asian immigrants are at particularly high risk for heavy drinking. Many of these immigrants left their homelands during or after the Vietnam War and, according to the author, their heavy alcohol use may result, at least in part, from war-related psychological problems. (pp. 270–275)

## SPECIAL POPULATIONS IN ALCOHOLICS ANONYMOUS

Alcoholics Anonymous (AA) is a self-help program first begun by white, Protestant, middle-class Americans. Drs. J. Scott Tonigan, Gerard J. Connors, and William R. Miller show that despite its narrow origin, AA is equally attractive to clients from a wide range of ethnic and cultural backgrounds. Preliminary results of the Project MATCH treatment study indicate that AA attendance may vary among clients with different backgrounds. For example, in some study groups the AA attendance rates of Hispanics and African-Americans were lower than those of whites. However, study findings also suggest that Hispanics show higher levels of commitment to AA than do whites, despite lower attendance. Furthermore, both AA attendance and involvement appear to be associated with improved abstinence regardless of racial/ethnic background. (pp. 281–285)

